# Supplementary material for: Are carbon emissions trading and green financial instruments synergistic? -Comprehensive quantitative research based on content analysis
Source: PLoS One. 2024 Mar 7;19(3):e0298601. doi: 10.1371/journal.pone.0298601 (PMC10919601; doi:10.1371/journal.pone.0298601)
Supplement: S1 Data — (ZIP) [file pone.0298601.s001.zip › Data/地级市Z_F工作报告.zip!/║┌┴·╜¡/╦1⁄2╤╝╔╜╩╨/└┤╫╘╣1⁄2╓┌║┼í╛┬φ┐╦╩2╛▌═°í┐_2019.docx]

各位代表：

现在，我代表市人民政府，向大会报告工作，请予审议。请各位政协委员和其他列席人员提出意见。

一、2018年工作回顾

2018年，市政府全面贯彻习近平新时代中国特色社会主义思想和党的十九大精神、习近平总书记在深入推进东北振兴座谈会上的重要讲话和考察我省的重要指示精神，全面落实十二届省委三次、四次全会和十一届市委二次、三次全会精神，在市委的坚强领导下，在市人大及其常委会和市政协的监督支持下，紧扣转型发展主题，着力推进高质量发展，经济社会运行总体平稳、稳中向好。预计全市地区生产总值增长5.2%，为五年来最高；规模以上工业增加值增长4.5%；固定资产投资增长5.5%；公共财政预算收入增长14.9%；社会消费品零售总额增长7%；城镇和农村常住居民人均可支配收入分别增长7%和7.5%；单位GDP能耗下降3.5%，较好地完成了市十六届人大二次会议确定的目标任务。

（一）转方式调结构取得重要进展。加快推进产业转型升级，培育壮大经济新动能。接续替代产业不断壮大。国能宝清2×60万千瓦电厂、鸿展30万吨特优食用酒精、万里润达150万吨玉米深加工等一批“四头四尾”和“七大新增长领域”项目加快推进，玉米深加工产业集群初步形成。全市开复工500万元以上产业项目126个（其中5000万元以上项目45个，亿元以上31个），竣工40个，完成投资59.7亿元。加快提升园区产业承载和集聚能力，市经开区固定资产投资增长52.9%。工业企业效益得到改善。推进78个已竣工项目实现投产达产。重点监测的22户企业各项生产效益指标均呈两位数增长。建龙钢铁、双矿两户骨干企业利税分别增长120%和21%。现代服务业健康发展。交通物流业加快发展，铁路发货量、快递业务量分别增长18.6%和38.8%。开辟精品旅游线路10条，建设圣洁·摇篮山公园等重点旅游项目11个，旅游业收入增长30%。电商企业发展到538户，增长26.6%。黑龙江“众农联”产业互联网平台型总部经济项目上线运营，线上交易额突破60亿元。金融产业持续发展，全市银行业贷款余额913.8亿元，增长5.2%；累放贷款450亿元，存贷比104.3%，高出全省平均水平21.6个百分点。深入推进以对俄为重点的对外开放，预计实现外贸进出口总值10亿元，同比增长4.8%。新的发展动能正在集聚。与高校、科研院所建立大豆、富硒产品等产业技术创新联盟15个，推广应用科技成果46项；实施重点科技成果转化和科技研发项目28个，新增科技型中小企业30家。东北唯一水稻种植业研究中心—中国水稻研究所北方水稻研究中心在宝清县正式成立。大力推进“两山合作”，成功举办了双鸭山经贸合作企业家恳谈暨“两山合作”经贸交流会，米高8万吨高效钾肥项目一期竣工投产，双山全民健身中心等7个民生项目加快推进。

（二）乡村振兴取得明显成效。深化农业供给侧结构性改革，着力提升农业农村发展质量。特色农业持续发展。粮食生产实现“十五连丰”。杂粮杂豆和经济作物分别达到14.5万亩和25.4万亩。棚室蔬菜增至1.1万亩，食用菌3514万袋，林业产业总面积达到14.3万亩。绿色生态农业加快发展。实施农业“三减”99万亩。绿色、有机食品认证面积增长到140万亩，“三品一标”认证产品达到360个，国家农产品地理标志认证达到11个。农业基础设施不断加强。“互联网+农业”高标准示范基地达到52个，增加6个。质量可追溯面积达到314万亩。新增高效节水灌溉面积1万亩。农村改革加快推进。农民专业合作社发展到1675个，家庭农场580个。全市421个行政村清产核资工作全面完成，农村集体产权制度改革完成年度任务。加快农村土地“三权分置”改革，基本完成农村土地确权。农村人居环境整治成效明显。新建农村公路54.6公里，新建安全饮水工程126处，新建农村室内厕所2402户。“大棚房”问题专项清理整治扎实推进。非洲猪瘟疫情得到有效封堵。

（三）“三大攻坚战”取得阶段性成果。聚焦发力，攻坚推进。重大风险得到有效防范化解。严厉打击逃废银行债务、非法集资，全市银行业不良贷款率3.7%，比年初下降0.65个百分点。面对政府债务进入偿债高峰期的极为严峻考验，顶住压力、排除万难、倾尽全力，争取各方面支持，采取一切可能的方式，有效化解了巨额债务。精准脱贫成效显著。坚持“六个精准”，全面落实“两不愁、三保障”政策，狠抓贫困村“三通三有”建设，突出抓好产业扶贫，强化“智贫双扶”。饶河县顺利摘掉国贫县帽子，全市55个贫困村中剩余的17个全部出列；实现脱贫人口1360人。污染防治扎实推进。全力做好中央环保督察“回头看”反馈意见整改工作，全面建立和实行生态环境保护“五个一”工作机制。完成了虹焱热电超标排放整改，国电、大唐实现超低排放，淘汰建成区10蒸吨及以下燃煤锅炉28台，建龙干熄焦项目投入运行。秸秆实现全域禁烧、全时段禁烧。全力推进秸秆综合利用，落地秸秆“五化”项目32个。全市空气质量达标率92.9%，同比提高2.5个百分点，创历史新高。严格落实河湖长制，突出抓好水环境污染治理，安邦河治理六大类28项任务已完成19项。寒葱沟水源地和21处地下水水源地环境问题已全部完成整改。主城区和集贤县雨污分流工程基本完成。完成11处存量垃圾场治理。完成植树造林4.78万亩、退耕还湿1.1万亩。完成自然保护区问题整改114处。

（四）重要领域改革取得重大突破。全面落实重要领域改革攻坚任务，着力破除体制机制弊端。供给侧结构性改革成效显著。建立“一图一表一台账一方案”，坚持“四个一批”，引导企业算好“三笔账”，采取“十项措施”，把好“三道关”，淘汰关闭地方小煤矿41处，退出煤炭产能252万吨。完成房地产去库存5439套，粮食去库存240余万吨。落实减税降费政策，全年减免、缓征税款20亿元。“放管服”改革持续深化。市本级行政权力事项和行政责任事项分别减少23%和22.5%。商事登记实现“三十五证合一”。全面完成了国、地税征管体制改革和不动产登记机构集成模式改革。“一网、一门、一次”改革顺利推进，网上可办率、进厅率、“一窗”受理率和“最多跑一次”比例大幅提升。政府机构和事业单位改革加快推进。优化政府机构设置和职能配置，市县（区）政府机构改革同步推进。事业单位改革完成阶段性目标。国有企业改革全面启动。13户国有企业公司制改革已全面完成，国有林场改革通过省级验收。金融体制改革务实推进。四个县农信社已完成改制，市区联社改制进展顺利。省鑫正投资担保集团双鸭山分公司成立并投入运营。

（五）民生持续改善。着力提升人民群众的获得感、幸福感、安全感。全市民生支出占一般公共预算支出的比重高达84.3%。重大交通基础设施建设加快推进。牡佳客专双鸭山段施工顺利。友谊至集贤公路、集贤镇至福利镇公路加快建设，饶河口岸至大岱林场公路竣工通车，尖山至宝山公路实现简易通车。市政基础设施短板加快补齐。建设路立交桥、中心站立交桥加快建设。人民群众高度关注的康泰路建成通车，完成电大路、窑地路等5条干道、8条人行道和54条主城区背街巷道及交通微循环改造。完成51.6公里老旧供热管网、15.9公里供水管网和1.5万户老旧楼房室内供水管网改造。新建改造城市燃气管网42.5公里，完成5万户居民天然气置换。6个独立工矿区和4个区本部“两供一业”综合改造加快推进。新、改建城区水冲公厕28座。人居条件不断改善。全面完成采沉区棚改三年治理任务。城市棚改安置居民7107户，采沉区避险建设住宅3489套。解决了3.2万户居民多年不能办理不动产登记证的难题。改造住宅小区30个，主城区15年以上老旧小区全部完成改造，物业弃管小区和单体楼实现兜底服务。拆除违法违章建筑近3万平方米。就业和社会保障水平显著提高。城镇新增就业2.46万人，城镇登记失业率控制在3.9%以内。城乡居民基础养老保险待遇标准由每人每月90元提高到108元，医保最高支付限额达到28万元。在全国范围内实现了异地就医住院费用直接结算。市、矿医院医保结算实现双向开放。城市低保标准提高到每月556元，农村低保标准提高到每年3900元。累计发放各类救助资金4.7亿元，惠及群众31.5万人次。公共服务事业加快发展。我市被确定为全国第三批居家和社区养老服务改革试点城市，已完成20个居家和社区养老服务中心改造。市第二中学、第四中学投入使用，市第三十二中学完成主体工程，新、改建公办幼儿园5所。市妇幼保健院改造、口腔医院搬迁已完成。市图书馆新馆主体工程完工。东部山体市民公园二期工程已完成，岭东南湖冬泳训练基地成功承办了国际赛事。社会治理持续加强。深刻吸取“1·23”和“5·20”事故教训，全力抓好以煤矿为重点的安全生产工作，生产安全事故死亡人数同比下降28.6%。严格落实信访责任制，扎实推进社会矛盾纠纷化解。深入开展“扫黑除恶”专项斗争，打掉黑恶势力19个，破获案件104起，治安案件和刑事案件发案率实现双下降。在全省公安机关缉捕网上在逃人员“百日攻坚”专项行动中，位列第一。创建全国禁毒示范城市任务全面完成。

（六）政府服务效能得到加强。深化机关作风整顿，大力优化营商环境，全力推进政府失信违诺、司法执法环境、不作为乱作为、公职人员勾结“黑中介”专项整治，共查处破坏营商环境典型案件50件，曝光典型问题45个。严格落实党风廉政建设责任制，强化“一岗双责”。狠抓中央巡视和省委巡视问题整改，市政府负责的任务全部按照时间节点有序推进。制定完善了《市政府工作规则》等11项制度，推进依法、科学和民主决策，决策“三重一大”事项101项，对78项重大事项进行了合法性审查。各级政府全部建立了首席法律顾问制度。市政府自觉接受市人大的法律监督和市政协的民主监督，办理人大代表建议、政协委员提案146件，办复率、满意率均达到100%。加大政务公开力度，累计公开政务信息1.5万余条。举办“双鸭山发展讲坛”12场，着力提高政府系统干部和公职人员工作专业化水平。

国防动员、双拥工作、统计监测、审计工作、外事侨务、民族宗教、档案史志、防灾减灾、地震气象等各项工作及妇女儿童、老龄和残疾人事业都取得了新的进展。

各位代表！过去的一年，我们坚决贯彻落实党中央国务院、省委省政府和市委各项决策部署，解决了一些历史遗留问题，完成了一些复杂任务，办成了一些涉及老百姓切身利益的实事。这些成绩的取得，无不得益于省委省政府和市委的坚强领导，无不得益于市人大、市政协的有效监督和社会各界的鼎力支持，无不得益于全市上下的顽强拼搏、实干苦干。在此，我代表市人民政府，向全市人民，向各级人大代表、政协委员，向各民主党派、工商联、无党派人士和人民团体，向驻双解放军、武警官兵、公安干警和消防救援队伍指战员，向在双中省直单位和所有关心支持双鸭山发展的各界人士，表示衷心的感谢和崇高的敬意！

同时，我们也清醒地看到，在发展中依然面临着诸多问题和挑战。主要是：经济总量小、发展速度慢、质量效益不高的问题依然突出;体制机制不活、市场化程度不高、民营经济发展不充分，深化改革的任务依然艰巨；城市建设和民生领域欠账较多，还不能很好地满足人民群众对美好生活的需要；部分政府系统干部专业化水平和担当精神还有明显差距;营商环境有待进一步优化。但我们更应该看到面临的重大机遇：一是习近平总书记这次到东北、到我省考察，给我市转型发展带来了重大政治机遇、环境机遇、发展机遇；二是即将出台的国家支持东北新一轮振兴的政策和我省支持煤城转型的政策，将为我市转型发展提供强有力的政策支持；三是全市上下精神振奋、干劲十足、力量凝聚。我们坚信，只要全市人民心往一处想、劲往一处使，抢抓机遇、迎接挑战，解放思想、聚力攻坚，就一定能够在转型发展的道路上迈出坚实的步伐，就一定能够推动双鸭山全面振兴全方位振兴。

二、2019年主要任务

2019年，是新中国成立70周年，是全面建成小康社会的关键之年，是推进全面振兴全方位振兴承上启下的开局之年。做好2019年政府工作至关重要。我们要按照市委的部署，以习近平新时代中国特色社会主义思想为指导，全面贯彻党的十九大精神和中央经济工作会议精神，聚焦落实习近平总书记在深入推进东北振兴座谈会上的重要讲话和考察我省的重要指示精神，切实贯彻省委十二届四次全会精神和省委经济工作会议精神、市委十一届三次全会和市委经济工作会议精神，统筹推进“五位一体”总体布局，协调推进“四个全面”战略布局，坚持稳中求进工作总基调、坚持推进高质量发展，坚持新发展理念，坚持以供给侧结构性改革为主线，坚持深化市场化改革、扩大高水平开放，坚决打好“三大攻坚战”，统筹推进稳增长、促改革、调结构、惠民生、防风险，落实“六稳”要求，聚焦实现双鸭山全面振兴全方位振兴“十个新”目标，坚定不移走好“六条路径”，深入解放思想，提振市场信心，激发内生动力，全面加强党对经济工作的领导，为决胜全面建成小康社会、实现双鸭山全面振兴全方位振兴打牢坚实基础，以优异成绩庆祝新中国成立70周年。综合考虑各方面情况，2019年发展主要预期目标是：全市地区生产总值增长5.5%；规模以上工业增加值增长5%；固定资产投资增长7%；公共财政预算收入增长6%；外贸进出口总值增长10%；社会消费品零售总额增长7.5%；城镇和农村常住居民人均可支配收入均增长7%。

各位代表，做好今年各项工作，要求我们必须把习近平总书记在深入推进东北振兴座谈会上的重要讲话和考察我省的重要指示精神作为根本遵循和行动指南，必须把中央提出的“巩固、增强、提升、畅通”八字方针贯穿于经济发展的各个方面和全过程，必须把加快推动一二三产同向发力的全面振兴作为主攻方向，必须把国家赋予龙江的战略定位与我市资源禀赋、区位条件、产业基础等优势结合起来的全方位振兴作为使命担当，加快走出一条质量更高、效益更好、结构更优、优势充分释放的振兴发展新路。

（一）着力推进产业高质量发展。把重点放在推动产业转型升级上，把发展壮大接续替代产业作为核心任务。强化产业项目支撑。实施新一轮产业项目三年行动计划，树立大抓项目的工作导向，围绕做实“四头四尾”、做强“七大新增长领域”和做深“五大产业链条”，大上项目、上大项目，以项目建设稳增长、稳投资、稳就业。重点推动鸿展和万里润达玉米燃料乙醇项目完成主体建设，中双石墨采选、东晟生态农业循环、四排风情小镇等项目加快建设，乌苏里船歌百里黄金旅游带、松江文化旅游商业街等项目开工建设，加快60万吨煤制烯烃、国能宝清二期2×100万千瓦超超临界机组等项目前期工作，完成国能20万吨腐殖酸褐煤蜡项目中试。层层建立项目储备库，抓紧谋划一批大项目，力争进入全省“百大项目”集群。全年开复工500万元以上产业项目150个（其中5000万元以上产业项目35个、2亿元以上省重点产业项目13个），竣工45个，完成投资70亿元；谋划储备5000万元以上产业项目60个（其中2亿元以上省重点产业项目13个）。提升招商引资实效。把招商引资作为经济工作的“生命线”。在招商引资中树立“舍得”理念，落实“四个一”工作机制，实施重点项目分级管理制度，建立“专班+园区”“目标+考核”的招商引资新机制，开通重点项目审批绿色通道，制定出台更优惠的招商引资奖励政策，摸清优势资源底数，成立招商专班，明确招商目标，落实招商责任，创新招商方式。全力推动国信通和中铝项目谈判，力求取得实质进展。提升园区承载力。把园区作为促进产业集聚、推动高质量发展的重要载体。制定出台促进开发区改革和创新发展实施意见，进一步理顺开发区体制机制，向园区精准放权。高起点、高标准编制园区总体规划及专项规划，推动公共基础设施和重点项目向园区集聚，尽快产生投资倍增效应。把宝清煤电化（材）产业园区建设作为头号工程，强力推进，强化水、电、气、运等要素供给，为大项目入驻创造先决条件。市经开区、省级开发区项目集中区分别实现“九通一平”“七通一平”。提升工业企业发展质量。深入实施规上企业培育专项行动，大力推进竣工项目投达产，对市场前景及成长性好的企业进行重点扶持，加大亏损企业帮扶，降低亏损面。支持建龙钢铁做大做强，加快技术创新和产能优化。支持国电、大唐等电力企业发展。加快发展现代服务业。完善政策措施，推动金融、物流、健康养老、信息服务、农业支撑服务等现代服务业发展质量提升。以打造“中国·黑土湿地之都湿地旅游带”和“乌苏里江·界江风情旅游带”为牵动，着力在发展黑土湿地旅游和乌苏里江风情旅游新业态上实现新进展，旅游收入增长30%以上。坚持“模式升级、发展加速、产业壮大”的思路，加快推进电商产业提档升级，电商平台、电商企业分别发展到15个以上和600户以上。大力推动科技创新。分类制定政策措施，力争在建立研发机构、培育科技型企业、打造产业技术创新联盟、加快企业技术改造、建设孵化器（众创空间）、挖掘能源职院潜力和建设创新人才队伍“七个抓手”上实现重大突破。全市规上生产加工型企业建立研发机构比例达到10%以上，规上工业科技型企业实现全覆盖；新增科技型中小企业40家以上、国家高新技术企业2家；产业技术创新联盟达到18个，新推广应用科技成果50项以上；新实施重点科技成果转化和科技研发项目30个以上；科技企业孵化器实现县（区）全覆盖，申报省级以上孵化器2家。实施“头雁行动”，对优秀人才给予平台、资金、政策、环境支持，以头雁带雁阵，打破人才瓶颈制约。推进军民融合发展。大力推动“互联网+”、数字城市和智慧城市建设。完善数字双鸭山和智慧双鸭山顶层设计，推动智慧城市一期各系统上线，推进建龙钢铁、黑龙新双锅等企业信息化、数据化、智能化，支持黑龙江“众农联”等项目取得新突破，加快数字化和人工智能技术与实体经济深度融合，推动新经济、新业态、新模式发展壮大。

（二）着力推进农业农村现代化。以深入实施乡村振兴战略为总抓手，提升农业农村发展质量。着力优化种植结构。聚焦“六个围绕”调结构，增加大豆种植面积，扩大杂粮杂豆和鲜食玉米、果蔬等特色经济作物种植面积。大力发展食用菌、林业产业、棚室蔬菜等特色种植产业。加强中药材基地建设，发展中药材产业。完成耕地轮作面积27万亩、休耕面积10万亩。因地制宜发展畜牧业。加快发展绿色农业。巩固绿色发展优势，农业“三减”面积扩大到120万亩。强化“三品一标”质量监管，绿色、有机规范化认证主体数量达到48个。加强农业基础设施建设。推进高效节水灌溉和高标准农田建设。农机作业综合机械化程度达到97%。强化农产品市场营销。以“宝清大米”“饶河东北黑蜂”等地标产品为核心，加大品牌整合、创建和推介力度，加快培育一批特色明显、品质优良的地域品牌，对接广东等高端消费市场，农产品销售量增长10%以上。加快农村一二三产业融合发展。以农产品加工企业、新型农业产业模式和新型经营主体为牵动，促进农业与加工、电商、旅游、康养等产业深度融合，加快延长产业链、提升价值链。深化农业农村改革。建立小农户和现代农业发展有机衔接机制，把农民合作社、家庭农场等引入现代农业发展大格局。大力培育综合合作社、营销合作社，引导合作社由单一生产型向产加销一体化经营型转变。全面完成农村土地“三权分置”改革，土地规模经营面积稳定在260万亩以上。基本完成农村集体产权制度改革。

（三）着力深化重要领域改革。深入落实习近平总书记“五个有利于”要求，完善改革思路，做实改革措施，提高改革效能。深入推进供给侧结构性改革。认真贯彻落实“巩固、增强、提升、畅通”八字方针，重点在破、立、降上下功夫。稳步推进企业优胜劣汰，出清“僵尸”企业2户。加大煤炭行业淘汰落后产能化解过剩产能力度，加快保留的31个煤矿改造升级，严格标准，对整合关闭矿井严把“四关”，坚决做到真管真关真淘汰。加大房地产、粮食去库存力度，商品房库存去化周期控制在18个月以下，政策性粮食去库存260万吨。加快政府机构和事业单位改革。科学制定政府部门“三定”方案，实行大科室制改革，确保按时完成市、县（区）政府机构改革。坚持政事分开、事企分开、管办分离，加快事业单位分类改革。加快国资国企改革。启动混合所有制改革试点，吸引民营资本和社会资本参与国企改革，增强企业市场竞争力。加强市直党政机关和事业单位经营性国有资产集中统一监管。做好农垦、森工、双矿行政职能和办社会职能接收工作。深化金融体制改革。鼓励和支持金融机构创新金融产品，扩大信贷规模，推动新增贷款向民营企业、中小微企业倾斜，增强金融服务实体经济能力。加大对非法集资、互联网金融等问题治理力度，从严管控金融风险。持续发力化解政府债务风险，坚决遏制违规新增债务。激发民营经济发展活力。不折不扣落实国家和省支持民营经济发展政策，出台我市支持民营经济发展的若干意见，设立小微企业信贷风险补偿金、企业转贷周转金和政府产业引导基金，落实新入规企业奖励政策，激发企业家精神，鼓励社会资本参与PPP项目，民营经济增加值增长5%以上。

（四）着力提升开放合作水平。向开放合作要动能、要发展、要效益。扩大对外开放。按照省委“一窗四区”部署，抓住同江中俄铁路大桥今年通车的机遇，以发展跨境物流、跨境电商为纽带，加快进出口加工基地和物流基地建设，推动以对俄为重点的对外开放转型升级。加快饶河口岸货物查验处理区、饶河锦隆物流保鲜库等项目建设，做优宝清白瓜籽大市场、岭东花菇园区等进出口基地。饶河口岸过货量、过客量分别增长8%和5%。深化对口合作。全面深化“两山合作”“双深合作”，力争在招商引资、共建产业园区和重大项目合作等方面实现新突破。推进百冠科技8万吨不锈钢及科技馆等项目竣工，力争合作项目达到20个以上。大力发展县域经济。依托资源禀赋和现实基础，突出抓住特色经济、“拳头”经济、民营经济，加快构建“一个县一个主导产业”或“一个县两个主导产业”的县域经济发展新格局。

（五）着力推进生态文明建设。全面贯彻落实习近平生态文明思想，坚决打好污染防治攻坚战。打好蓝天保卫战。加大对钢铁等重点排污行业管控力度，加快建龙钢铁焦炉无组织废气排放和除尘设备改造。启动城镇建成区10蒸吨及以下燃煤锅炉淘汰工作。加快秸秆“五化”综合利用项目建设，秸秆综合利用率达到70%以上。推进秸秆全域、全时段禁烧，强化重污染天气联防联控联治，全年空气质量优良天数保持在330天以上。打好碧水保卫战。严格落实河湖长制，深入开展“清四乱”专项行动，加大地下水压采力度，加快“引松入双”工程前期工作。加快安邦河、挠力河流域水环境综合整治等工程建设，安邦河、挠力河国控断面分别稳定达到Ⅳ类和Ⅲ类水体标准。开展市二水源、寒葱沟水源地保护区农村生活污水专项治理行动。加快推进宝山区污水处理厂前期工作，确保市经开区等5座污水处理厂建成并投入使用。打好净土保卫战。全面实施土壤污染防治行动，建立耕地土壤环境质量清单、建设用地土壤污染风险管控和修复名录。实施黑土地保护专项行动，推进东北黑土区侵蚀沟综合治理工程建设。实施城镇垃圾处置能力提升行动，开工建设生活垃圾焚烧发电项目，启动餐厨垃圾处理工程。严格管理涉重金属企业，建立污染源排查清单。打好原生态保卫战。严格生态环境质量管理，确定并严守生态保护红线、环境质量底线、资源利用上线，制定生态环境准入清单。加大退耕还林、退耕还湿力度，完成退耕还林60公顷、还湿104公顷。打好美丽乡村保卫战。深入推进农村人居环境整治三年行动，建立县（区）、乡（镇）、村三级农村人居环境治理工作体系和责任体系。探索建立符合农村实际的生活垃圾处理机制，全市50%以上的行政村生活垃圾得到治理。启动城中村污水管网和4座重点镇污水处理厂建设。改造农村危房2455户，改建农村室内厕所5541座。抓好规模养殖场粪污处理设施装备配套工作，配套率达到80%以上。

（六）着力补齐城市建设短板。坚持规划先行，编制完成城市总体规划和“多规合一”空间规划及专项规划。抢抓国家加大基础设施领域补短板的机遇，争取落实实施一批工程和项目，扩大有效投资。以老城区为重点，实施城市建设“七大工程”：实施城区道桥工程。确保建设路立交桥7月竣工通车，力争中心站立交桥年底通车。继续实施主城区“疮疤”道路维护改造、背街巷道综合整治和交通微循环工程。实施老旧小区修缮改造工程。修缮改造主城区41个小区409栋住宅楼。全面完成6个独立工矿区和4个区本部“两供一业”综合改造。实施城市管网工程。完成15座供水二次加压泵房改造、新建改造供热管网20.1公里、供水管网38公里、燃气管网37公里。加大天然气置换力度，确保完成2万户居民天然气置换。推动“俄气入双”工程尽快实施。实施绿化美化工程。完成东部山体公园三期建设和北秀公园、和谐公园、益寿山公园维修改造。实施北秀公园西门等景观带及绿地建设，对九阳路等8处重点街道、广场绿化进行完善改造。开展“小开荒”整治，进一步增加城市绿地空间。实施公共服务工程。第三十二中学建成投入使用，新、改扩建幼儿园3所，市图书馆新馆全面建成。推进建设路市民健身广场、天力小区西侧停车场等一批项目建设改造。推进城区“厕所革命”，新建8座水冲公厕。实施城市精细化管理工程。加大城市管理综合执法力度，深化违法违章建筑治理，突出抓好市容市貌管理，推动“干净、整洁、有序、安全”城市建设。实施综合交通畅通工程。加快牡佳客专双鸭山段建设，推进宝迎铁路开工，力争高铁综合客运枢纽开工建设。加快友谊至集贤公路、集贤镇至福利镇公路、福利镇至太保镇公路建设，力争大岱林场至红旗岭公路开工，推进宝山至宝清公路开工，尖山至宝山公路竣工通车。加快“四好”农村道路建设，行政村通村路硬化率达到100%。

（七）着力优化营商环境。着力打造便捷高效的政务环境、优惠宽松的政策环境、公平正义的法治环境、崇德守信的人文环境、绿色宜居的生态环境。深化“放管服”改革。大力简政放权，以“放”为原则，“不放”为特例。全面实行“证照分离”，推进市场主体登记全程电子化。全市企业开办时间压缩到8个工作日以内，不动产登记5日内办结。加大“一网、一门、一次”改革力度，做到大多数事项“网上办”，必要到窗口的“一次办”，少数企业特殊事项由政府提供“我帮办”。深化“双随机、一公开”市场监管。探索企业投资项目承诺制。落实便利办税措施。以“办事不求人”为目标，开展营商环境评价。大力降低企业成本负担。降低企业制度性交易成本、税费成本、要素成本，依靠改革降低企业用能、用地、物流、融资成本。持之以恒深化机关作风整顿优化营商环境。建立和完善营商环境制度体系、监督体系、考核评价体系和大数据中心，强力推进失信违诺、司法执法环境、不作为乱作为、公职人员勾结“黑中介”等专项整治，严厉查处各类破坏营商环境行为，大力选树正面典型，狠刹歪风邪气，弘扬清风正气，着力构建“亲清”新型政商关系，对失信企业实行信用“黑名单”制，以良好的营商环境聚人聚财聚动能。

（八）着力保障和改善民生。无论遇到什么困难，民生保障都不能打折扣，严格控制和压减一般性支出，把财政资金更多地用在保障民生上。全力打赢精准脱贫攻坚战。着力抓好饶河县脱贫摘帽巩固提升和非贫困县、非贫困村脱贫攻坚。探索解决收入水平略高于建档立卡贫困户群体缺乏政策支持等问题。把产业扶贫作为根本之策，因村制宜、因户择业、因人施策。全面落实“两不愁、三保障”政策，着重解决因病因残致贫、住房保障和安全饮水问题。坚持“志智双扶”，建立正向激励机制。加强精准扶贫资金监管。完成剩余1240名贫困人口脱贫任务，实现农村贫困人口全部脱贫。扎实做好就业工作。落实积极的就业政策，加快公共就业服务体系建设，推进退役军人、高校毕业生、城镇登记失业人员和农村转移就业劳动者等群体多渠道就业。发挥大众创业万众创新对扩大就业的支撑作用，运用“互联网+”发展新就业形态。健全保障农民工工资支付长效机制。全年开展职业技能培训8000人次以上，实现城镇新增就业2.1万人、转移农村劳动力13万人。切实提升社会保障水平。提高城镇职工养老保险待遇，继续推进机关事业单位养老保险制度改革。全面实现省内异地就医门诊直接结算。完善医疗保险支付方式改革，提高医保基金使用效率。提高城乡居民医疗保险政府补贴标准。提高低保、特困保障标准，扩大覆盖面，提高精准度，保障下岗失业、就业困难等城市边缘群体基本生活。健全社会救助体系，切实保障城乡困难群众基本生活。持续改善人居条件。完成采沉区棚改配套基础设施建设，完成宝山区采沉区避险项目安置工作。大力发展公共服务事业。普惠性幼儿园覆盖率达到65%。确保宝山区义务教育基本均衡发展通过国家验收，科学配置矿区和农村学校教育资源。较高水平普及高中阶段教育，深化普通高中课程改革和高考改革。严肃整治教育“三乱”。深化产教融合、科教融合，把职教集团打造成科技创新基地和高技能人才培养基地。深化医药卫生体制改革，完善分级诊疗模式，加强全科医生队伍建设。严厉打击非法行医、非法执业行为，保障医疗安全。强化监管，确保食品药品安全。做好非洲猪瘟等重大动物疫病防控工作。抓好全国第三批居家和社区养老服务改革试点，启动智慧养老综合服务管理平台建设。完善公共文化服务体系建设和城乡文化设施布局，深入推进文化惠民工程，开展全民阅读活动。广泛开展全民健身行动，深入推进“三大球”和冰雪运动项目进校园，广泛开展群众性体育活动。积极发展妇女儿童、老龄和残疾人事业。创新完善国防动员体系，深化双拥共建，做好退役军人接收安置工作，落实好抚恤优待政策。以创建全国文明城市为牵动，着力提升全民文明素质，塑民风、树新风，引领社会文明风尚。

（九）着力加强和创新社会治理。全面提升社会治理社会化、法治化、智能化和专业化水平。全力抓好安全生产工作。严格落实安全生产属地管理、部门监管和企业主体责任，健全完善风险分级管控和隐患排查治理双重预防机制，严格落实“一项制度、三个清单”网格化常态化监管机制。突出抓好煤矿安全生产，全面开展从业人员“安全意识提升年”活动，加强煤矿现场兑规作业管理，严厉打击“三违”行为和违法违规生产行为。抓好非煤矿山、道路交通、建筑施工、危险化学品等各行业领域安全隐患排查整治，坚决遏制重特大事故发生，创建全省城市安全发展试点城市。全面加强应急管理，建立应急管理协调指挥联动机制，积极应对生产安全事故、自然灾害等突发事件，做好防灾减灾工作。深化“平安双鸭山”建设。深入实施公安系统“六大工程”，持续推进“龙防云”网络110平台和“雪亮工程”建设，强化“天眼工程”实战应用。科学调整市、县（区）巡逻防控力量布局，完善立体化、信息化治安防控体系。深入开展“扫黑除恶”专项斗争。扩大法律援助范围，为弱势群体维权。着力强化信访工作。严格落实信访工作责任制和“三访一包”工作机制，落实“三个到位、一个处理”要求，坚持“四个一批”治访模式，健全信访矛盾多元化解工作机制，全面落实社会矛盾化解“责任清单”和信访工作“负分制”考核办法，加大信访积案攻坚力度，努力从源头化解矛盾。

（十）着力加强政府自身建设。做好今年各项工作，必须进一步提高各级政府的创新力、执行力、公信力和凝聚力。持续深入开展“解放思想推动高质量发展大讨论”、“我为招商引资做贡献大讨论”，聚焦“七破七立”，以思想大解放、观念大更新，推动思路再创新、工作再抓实、改革再深入、发展再提升。健全依法决策机制，不断提高政府依法决策、科学决策、民主决策水平。坚持依法行政，严格遵守宪法和法律，提高运用法治思维和法治方式开展工作、解决问题、推动发展的能力。自觉接受人大法律监督、政协民主监督以及社会各界监督。推进政务公开，让权力在阳光下运行。推进严格规范公正文明执法。加强政府领导干部和公职人员业务能力培训，凭专业说话，按程序办事，做真懂经济、善抓经济的行家里手。建立容错、纠错、防错机制，激发干部担当作为。树立过紧日子思想，勤俭节约，强化重点资金和项目审计监管，把政府有限资金用在“刀刃上”。落实“一岗双责”，不断加强政府系统党风廉政建设和反腐败斗争。大力转变工作作风，力戒形式主义、官僚主义，落实“五细”要求，弘扬“五个好作风”，争做“三个好把式”，切实践行好“人民政府为人民”的根本宗旨。

各位代表，新的起点、新的征程、新的作为。市政府坚信，只要我们全面贯彻落实习近平新时代中国特色社会主义思想、习近平总书记在深入推进东北振兴座谈会上的重要讲话和考察我省的重要指示精神，牢固树立“四个意识”，做到“两个维护”，在市委的坚强领导下，解放思想、锐意创新，攻坚克难、奋发图强，就一定能够推动双鸭山高质量发展，就一定能够推动双鸭山加快转型，就一定能够推动双鸭山全面振兴全方位振兴！
